# Reducing surgical site infections (SSI) in breast surgeries, including a newly identified risk for sentinel node biopsies

**DOI:** 10.1186/2047-2994-4-S1-O31

**Published:** 2015-06-16

**Authors:** M Catlin, C Blue, R Thompson, A Nelson

**Affiliations:** 1 Infection Prevention and Employee Health, Group Health Cooperative, Seattle, WA, USA

## Introduction

In 2012, 19 infections out of 561 breast surgeries (Standardized infection Ratio (SIR) 3.372, 95% CI 2.09-5.168) led to the discovery that non-sterile radiation probes pierced their sterile sheaths. After disinfecting the probes, the SSI was 0%, then subsequently increased.

## Objectives

The objective of this study was to identify risk factors for continued infections in 2013 and 2014.

## Methods

Rates of SSI were calculated for each surgeon. Case control analyses identified risk factors using R version 3.1.2. Cases were observed and results shared with surgical teams.

## Results

Of 26 surgeons, Surgeon X had 33% (386) of the 1169 procedures in 2012-2013 and 52% (13/25) of the infections: the SSI rate was 3.38% (13/384), which was significantly greater than expected 0.32 from the U.S. National Health Safety Network risk adjusted control cases (SIR 3.117 ( 95% CI1.73-5.20)).

Surgeon X’s sentinel node biopsies in 2014 had a 9-fold increased SSI risk (OR 9.0, Fisher Exact, p=.051); several practice variations were observed.

## Conclusion

The rate of SSI was reduced to zero in the 4^th^ QTR of 2014, after communicating surgeon-specific rates, possible risk factors, and peer coaching. Practice changes included high-level disinfection (HLD) of radiation probes used in surgical fields, use of disposable hair bonnets, anchoring drains, and revised prepping.

## Disclosure of interest

None declared.

